# Using deep learning to capture gravel soil microstructure and hydraulic characteristics

**DOI:** 10.1038/s41598-025-04879-4

**Published:** 2025-07-01

**Authors:** Bin Zhu, Yu-Fei Xie, Xiang-Gang Hu, Dai-Rong Su

**Affiliations:** Earth Sciences College, Guilin University of Technology, Guilin, 541004 China

**Keywords:** WGANs (Wasserstein generative adversarial networks), Machine learning, Porous structure, Pore-scale CFD simulation, Hydraulic properties imitation, Hydrology, Hydrogeology, Sedimentology

## Abstract

The special hydraulic properties of gravel soil, attributed to its varying fine particle content, can be effectively analyzed using the Wasserstein Generative Adversarial Networks (WGANs) technique. This approach enables the reconstruction of 3D digital samples of gravel soil, allowing for the generation of specific microstructure realizations, including complex pore characteristics. This capability is crucial for gaining insights into the hydraulic behavior of gravel soil. In a specific case involving gravel soil samples from Guilin city, China, three samples with similar structural features were carefully selected for analysis. These samples were scanned using µ-CT to create the training dataset for the reconstruction model. The WGAN with Gradient Penalty technique was then applied to simulate the reconstruction of the digital gravel soil samples. The results demonstrated a high consistency between the reconstructed model of gravel soil realizations and the original samples in terms of porosity, two-point correlation function, linear path function, specific surface, and Euler characteristics number. Furthermore, through the evaluation of permeability, it was shown that the reconstructed realizations effectively captured and represented the actual soil prototype. This allowed for the analysis of seepage characteristics and internal stability within a range of magnitudes up to 10^− 2^ cm/s. Compared with the machine learning model generated in previous literature, the machine learning model recommended in this paper can capture the hydraulic properties of gravel soil with obvious difference in coarse and fine particle size, which is reflected in the difference of permeability of the two orders of magnitude.

## Introduction

Studying the internal microstructural characteristics of gravel soil is crucial for understanding the fluid flow patterns. In Guilin city, most constructions are located on the top of the II-III level terraces along the Li River. A special layer of gravel soil, consisting of pebble gravel intercalated with fine sand or silty clay, exists as the foundation soil layer. Due to its abundant groundwater, it is prone to perturbation and disturbance during engineering activities, which can lead to disasters such as seepage erosion, piping, soil flowing and even water-inrush^1^. Similar engineering challenges posed by uneven gravel particles are affecting not only Guilin but also engineering practice worldwide^2–4^. Obtaining intact gravel soil samples is challenging because sampling and laboratory recompaction are often costly and laborious. Furthermore, the distribution of hydraulic properties in naturally obtained specimens is challenging to control. Therefore, it is a difficult challenge to capture the micro-hydraulic characteristics of gravel soil.

To solve the above engineering problems, it is necessary to understand the internal structure and hydraulic performance of geotechnical materials. A set of techniques was proposed to acquire the digital images of gravel soil samples by using µ-CT scanning^5^ first. This approach involves sampling standard soil samples and obtaining their images through CT scanning. We then selected representative images as the training set for image generation to reconstruct virtual core samples, which have similar microstructure and properties to the original soil samples. Subsequently, the hydraulic properties and internal stability of the reconstructed samples are investigated and evaluated. The solution can reconstruct more digital 3D virtual samples that exhibit identical properties to the standard samples, which honor to the microstructure features of original samples collection. It is also the foundation of virtual geological scenarios, known as the digital-twin technique for material microstructures^6,7^. This approach assists in reducing reliance on extensive trial-and-error experiments.

Generative adversarial networks (GANs)^8^ technique is unquestionably the new favorite in the reconstruction or digital-twin trend with unsupervised intelligent deep learning approach, which is characterized by its ability to generate realistic data by two neural networks, the generator and the discriminator, working in opposition to each other. Therefore, GAN models can be used for data generation, data augmentation, sample expansion, etc., especially in situations where there is a lack of annotated sample data. Currently, GAN technology has been applied in various fields such as soil CO_2_ plume migration prediction^9^, 3D digital core reconstruction of rock mass, as described in previous literatures^11^. To overcome the problem of unstable training, Gulrajani et al. (2017)^12^ developed an improved algorithm so-called Wasserstein Generative Adversarial Networks with Gradient Penalty (WGAN-GP) to eliminate vanishing and exploding gradient problems.

Large scale spatial model reconstruction is generally to use the MPS-based (multiple-point statistics) methods to reconstruct stochastic random media, which have been used in two- and three-dimensional conditional simulations of spatial properties in reservoir-scale earth modeling applications^13,14^. MPS methods can reproduce complex geological structures and patterns that are characteristic of the earth’s subsurface, and capture the heterogeneity and uncertainty present in the subsurface. A geological modeling method integrating MPS and GANs was proposed by Laloy (2018)^15^ for the generation of river channels or reservoirs^16^. When integrated with MPS, GANs can further enhance the realism and diversity of the generated geological features, enabling the production of highly detailed and diverse river channels and reservoir structures. An earlier stochastic method, called simulated annealing, can be classified as an early intelligent training algorithm. It allows the incorporation of arbitrary cost functions of statistical and morphological properties used in unconditional three-dimensional image reconstruction^17,18^.

For the reconstruction of small- or pore-scale media, the fundamental approach involves using digital images obtained from three-dimensional microscopy instruments such as *µ*-CT or medical-CT instruments. Then using these digital images as training set, GAN technology is carried on to train and generate images of virtual soil samples with the same structural characteristics^19^. Comparing the existing GANs framework, the GAN, considering of gradient-differentiable Wasserstein distance as loss function, can overcome the limitations of KL (Kullback-Leibler) and JS (Jensen-Shannon) divergences, and provide a more reliable and comprehensive measure of dissimilarity between probability distributions. At the same time, the method of penalty function ensures more stable training performance^20^.

In recent years, significant progress has been made in studying soil hydro-mechanical behavior by integrating physical mechanisms with data-driven approaches. Li et al.^21^ developed a model for collapsible loess based on the wetting soil-water characteristic curve (SWCC) and Fredlund-Xing equation, demonstrating superior prediction accuracy compared to Chinese standard methods and highlighting the critical role of unsaturated soil mechanics in collapse assessment. Kim et al.^22^ employed a multilayer perceptron (MLP) model to predict the effective cohesion of residual soils in Singapore, combining it with Kriging analysis to examine spatial variability and explaining cohesion differences across geological formations through microstructural pore distribution characteristics. Tian et al.^23^ revealed that compaction state significantly influences clay desiccation cracking, with wet-dry cycles exacerbating crack network complexity. These studies showcase the advantages of machine learning in this field, while challenges remain in cross-scale mechanism interpretation, multi-source data fusion, and physical constraint embedding. Future research should focus on developing hybrid models that balance mechanistic interpretability with data-driven accuracy.

In the second section, three groups of gravel soil samples are prepared in the laboratory. The training set production process and WGAN-GP technology are also described in this section. In the third section, the images are then fed into the GAN model for training to obtain a well-trained generator model. The generator model is utilized to generate numerous digital image realizations. In the fourth section, they are subsequently evaluated by inspection index, such as MDS (multiple dimensional scaling) for visualizing generation process, and morphological descriptors for assessing similarity of the generated results to real samples, to obtain reconstructed samples that closely resemble real gravel soil samples, in which a comparison is conducted to assess the consistency of parameters such as porosity, two-point correlation function, and linear path function. In the fifth section, the porosity, specific surface, Euler number and permeability of generative gravel soil samples are compared to identify their hydraulic behaviors and erosional stability characteristics of the reconstructed samples. Some discussion and conclusion are presented in the sixth and the last section to verify the validity of the method proposed.

The aim of this paper is to propose a viable deep learning technique for reconstructing gravel soil digital samples that conform to specified particle size distributions and hydraulic behaviors.

## Materials and methods

### Material preparation and image acquisition

The materials for study were sourced from an open-pit excavation site along the banks of the Li River in Guilin, China. Each component of every specimen, including both the main and sub-groups, undergoes careful sieving, weighing, and is then placed into transparent acrylic containers for CT scanning. These materials are characterized by a deficiency in the median particle size, with a high abundance of both coarse and fine particles, which contributes to their distinct microstructure and permeability properties. Through detailed analysis, three distinct microstructural modes were identified, which are considered as training sets for machine learning.

**Fig. 1:**
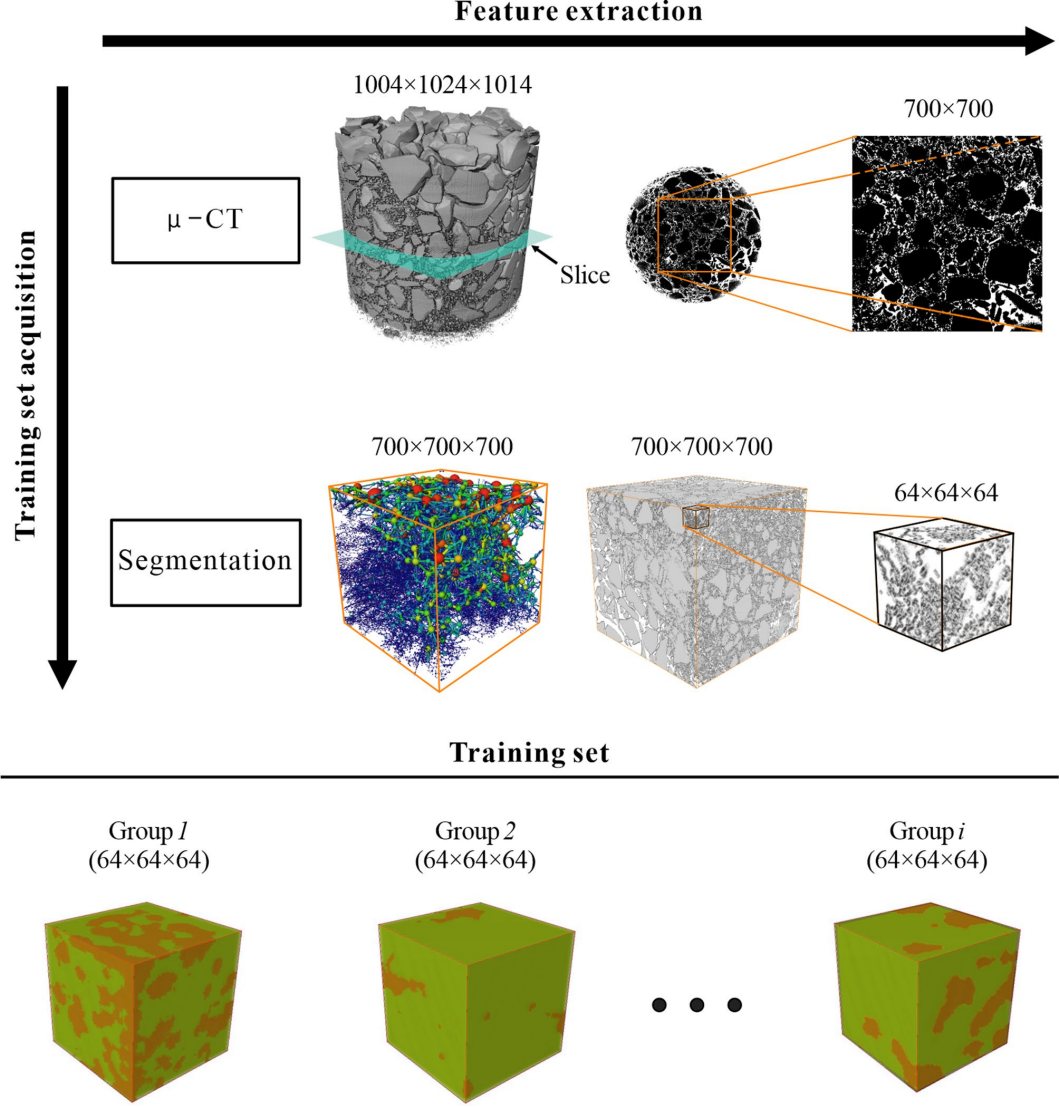
A scheme of randomly selected 64 × 64 × 64 training sets from 700 × 700 × 700 gravel soil prototypes with unique porous distributions.

The study encompassed three groups of granular soil specimens, consisting of both coarse and fine grains. The coarse grains had a size range of 2 mm to 10 mm, while the fine grains ranged from 0.065 mm to 2 mm, with cumulative fine grain contents varying between 30% and 34%. Each group of specimens exhibited a gap-graded gravel structure, with particles absent in the 1–2 mm, 0.5–1 mm, and 0.25–0.5 mm size fraction. These soil samples were packaged in transparent acrylic cylindrical containers with a height and diameter of 35 mm. To examine the internal structure and arrangement of particles, high-resolution 3D X-ray microimaging was performed using a Zeiss Xradia 510 Versa system. The CT imaging produced voxel datasets with dimensions of 1004 × 1014 × 1024, at a resolution of 35 μm/voxel, enabling detailed visualization of the soil’s particle distribution. The resulting 3D images (shown in Fig. 1) revealed a phenomenon where coarse particles appeared to “float” within the finer particles, providing insights into how different particle sizes interact in gap-graded soils. These findings offer valuable information for understanding the behavior and properties of granular soils in various engineering applications.

### Training sets acquisition

After the CT scanning process, a dataset of 1014 pieces of 2D CT images with dimensions of 1004 × 1024 pixels was obtained. The Threshold function is used to perform threshold segmentation on these images in ImageJ software. Setting different threshold values based on the grayscale intensity, the gravelly soil can be segmented into two distinct components: pores and soil skeleton. To extract images in bulk using the batch cropping function of ImageJ software, select regions with well-developed pores and crop them to a size of 700 × 700 pixels. Each set should consist of 700 cropped images. Overlaying 700 2D images to create a 3D image, the resulting 3D image can be sliced into multiple 64 × 64 × 64 *voxel*-sized 3D images. This process will yield three datasets, each consisting of 1900 training sets. The training sets are illustrated in Fig. 1.

By selecting training sets randomly from the primitive soil prototype, we can capture different characteristics of porous distribution within the gravel soil samples. Each group of training sets may exhibit unique patterns or arrangements of pores within the specified dimensions. This approach allows for the generation of diverse training data that represents the variability in porous distribution within the gravel soil samples. Analyzing the characteristics and distributions of these training sets can provide insights into the overall behavior and properties of the gravel soil samples.

The preprocessing of micro-computed tomography (µ-CT) images is a crucial step in preparing the data for advanced analysis, particularly when utilizing machine learning algorithms. Preprocessing steps improve the quality of µ-CT images by cleaning, aligning, and segmenting the data, reducing errors and enhancing machine learning model accuracy, allowing for deeper insights into material properties and ensuring high-quality data for advanced computational analysis^24^. The process typically begins with **median filtering**, which is used to eliminate noise, such as “salt-and-pepper” artifacts that can obscure critical details in the image. By replacing each pixel’s value with the median of its neighboring pixels, this technique effectively reduces random noise while preserving the sharpness and edges of important structures, which is vital for maintaining the integrity of the data. The next step involves **image alignment and registration**, which corrects any misalignments that may have occurred during the capture of multiple images. This is especially important when µ-CT scans are taken from different angles or at different times, as slight shifts or rotations can cause inconsistencies that make it difficult to analyze the images collectively. Image registration ensures that corresponding points across the images are aligned, making it possible to create a cohesive dataset for more accurate and reliable analysis. Following alignment, **thresholding segmentation** is applied to separate different regions of interest in the image based on intensity values. This process allows for the differentiation of solid materials from pores (empty spaces within the sample), which is essential for studying the material’s structure, porosity, and other relevant characteristics. A specific threshold is chosen to distinguish between high-intensity (solid) and low-intensity (porous) regions, enabling clearer identification of key features.

### Generative adversarial Nets (GANs)

**Fig. 2:**
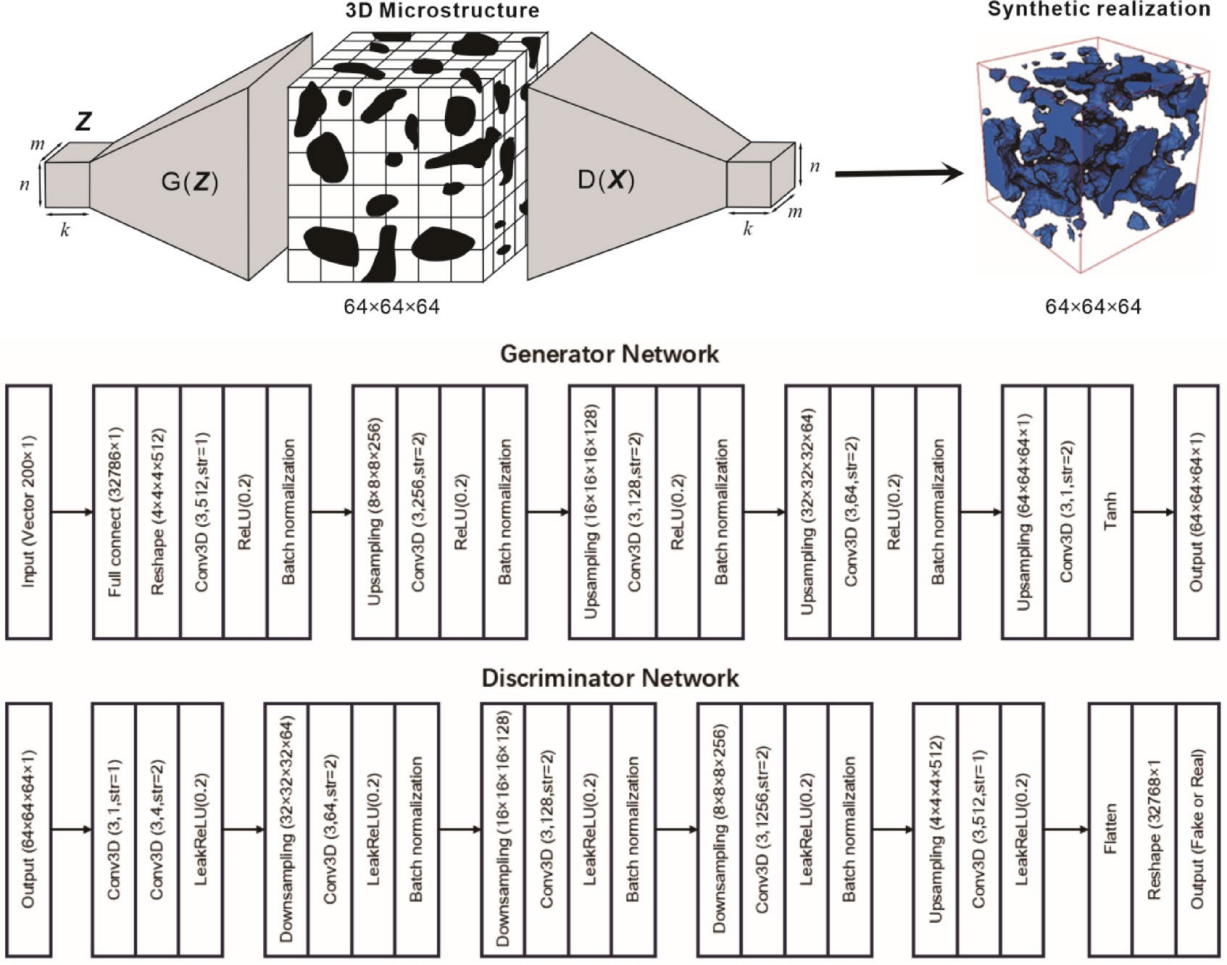
The architectures of the generator and the discriminator used in this study. The input of the generator is a 200-dimensional vector sampled from the Gaussian distribution, i.e., z ~ Gaussian (0, 1). The main body of GAN’s is a convolutional neural networks (CNNs) in the generator and discriminator, with strides and pooling layers for up-sampling and down-sampling. Wasserstein distance loss function is used to instead of binary cross-entropy loss, which provides more stable training.

Goodfellow et al.^8^ proposed the framework of GAN technique to address the generative problem, which aims to train a generative model that can approximate an unknown distribution *p*_*data*_ over a high-dimensional space *χ*, given many observed real samples *x*_*r*_ from this distribution, i.e., *x*_*r*_ ~ *p*_*data*_. Figure 2 illustrates the typical workflow of a GAN. A GAN generally consists of two trainable blocks, a generator (*G*_*θ*_) and a discriminator (*D*_*φ*_), with *θ* and *φ* being trainable parameters. A latent variable *Z* is defined with a known distribution *p*_*z*_ (e.g., Gaussian) over a low-dimensional space Ƶ, and *z* is considered a sample from Ƶ, i.e., *z* ~ *p*_*z*_. The generator *G*_*θ*_ maps *z* into *x*_*G*_, which lies in the space *χ*, i.e., *x*_*G*_ = *G*_*θ*_(*z*); the distribution of *x*_*G*_ is *p*_*G*_, i.e., *x*_*G*_ ~ *p*_*G*_. The discriminator *D*_*φ*_ maps the generated sample *x*_*G*_ and the given data sample *x*_*r*_ into two scalar values called scores, *s*_*G*_ and *s*_*r*_, respectively, i.e., *s*_*G*_ = *D*_*φ*_(*x*_*G*_), *s*_*r*_ = *D*_*φ*_(*x*_*r*_). These scores evaluate the similarity of the inputs of *x*_*G*_ and *x*_*r*_. The loss function of GANs is based on some type of distance between *s*_*G*_ and *s*_*r*_ (Eq. (1)), which represents the distance between *p*_*G*_ and *p*_*data*_. As shown in Fig. 2, the discriminator *D*_*φ*_ and the generator *G*_*θ*_ are alternatively trained by maximizing and minimizing the loss function (Eq. (1)) until a certain stopping (Minimax) criterion is reached.

1 $$\:{J}^{\left(D\right)}=-{\varvec{E}}_{{x}_{r}\sim{p}_{data}}log{D}_{\phi\:}\left({x}_{r}\right)-{\varvec{E}}_{{x}_{G}\sim{p}_{G}}log\left(1-{D}_{\phi\:}\left({G}_{\theta\:}\left(z\right)\right)\right)$$

2 $$\:{J}^{\left(G\right)}={\varvec{E}}_{{x}_{G}\sim{p}_{G}}log\left(1-{D}_{\phi\:}\left({x}_{G}\right)\right)$$

where *J* ^(·)^ is some type of distance between *x*_*G*_ and *x*_*r*_, such as Jensen-Shannon divergence; ***E*** is the expectation over *x*_G_ ~ *p*_*G*_ and *x*_*r*_ ~ *p*_*data*_.

In Generative Adversarial Networks (GANs), the generator and discriminator engage in a competitive process until the generated data becomes indistinguishable from real data, with both distributions nearly identical, reaching a Nash equilibrium at the discriminator loss’s saddle point. The discriminator’s optimization strategy can be expressed as follow.

3 $$\:{D}_{\phi\:}^{*}\left(x\right)=\frac{{p}_{data}\left({x}_{r}\right)}{{p}_{data}\left({x}_{r}\right)+{p}_{G}\left({x}_{G}\right)}$$

where *p*_*data*_ and *p*_*G*_​ represent the real and generated data distributions, respectively.

This ratio is estimated using supervised learning, which is a core approximation mechanism in GANs. When the discriminator fails to distinguish between real samples *x*_*r*​_ and generated samples *x*_*G*_​, it signals that the generator has effectively learned to replicate the real data distribution, i.e., *x*_*r*_ ∼ *p*_*data*_​, indicating the generator’s ability to produce realistic samples.

### Loss function of WGAN with gradient penalty (GP)

In order to overcome the problem of unstable training in image generation of original GANs, new improvements have been proposed by many scholars in recent years^21,22^. The better the discriminator is trained, the harder it is for the generator to produce images. Therefore, a strategy for optimizing loss functions by using the gradient-differentiable Wasserstein distance to replace KL and JS divergences was proposed by Arjovsky et al.^20^.

Proposed the Wasserstein loss function based on the Wasserstein distance between *p*_*data*_ and *p*_*G*_ shown in Eq. (4)

4 $$\:W\left({G}_{\theta\:},{D}_{\phi\:}\right)={E}_{{x}_{r}\sim{p}_{data}}{D}_{\phi\:}\left({x}_{r}\right)-{E}_{z\sim{p}_{z}}{D}_{\phi\:}\left({G}_{\theta\:}\left(z\right)\right)$$

where *D*_*φ*_ does not have the sigmoid function in the last layer and constrains the backpropagate training parameters in the range of (-c, c) by the weight clipping.

Gulrajani et al.^12^ improved the clipping weight furtherly by an alternative way to penalizing the norm of gradient of the discriminator with respect to its input. Proposed method performs better than standard WGAN and enables stable training of a wide variety of GAN architectures with almost no hyperparameter tuning. This improved loss function is shown in Eq. (5)

5 $$\:W\left({G}_{\theta\:},{D}_{\phi\:}\right)={E}_{{x}_{r}\sim{p}_{data}}{D}_{\phi\:}\left({x}_{r}\right)-{E}_{z\sim{p}_{z}}{D}_{\phi\:}\left({G}_{\theta\:}\left(z\right)\right)-\lambda\:{E}_{\widehat{x}\sim{p}_{\widehat{x}}}\left[{\left({\|{\nabla\:}_{\widehat{x}}{D}_{\phi\:}\left(\widehat{x}\right)\|}_{2}-1\right)}^{2}\right]$$

where *λ* is penalty coefficient and default value 10; $\:{p}_{\widehat{x}}$ is the distribution of the sampled $\:\widehat{x}$ between *x*_G_ ~ *p*_*G*_ and *x*_*r*_ ~ *p*_*data*_ by the *t ~ uniform* (0,1) function, i.e., $\:\widehat{x}=t{x}_{r}+(1-t){x}_{G}$.

In our study, we will take the loss function of WGAN with gradient penalty as the minimizing objective for the backward propagation to ensure more stable training performance.

## Results

### Generation effects of 3-D models

#### 3-D models

**Fig. 3:**
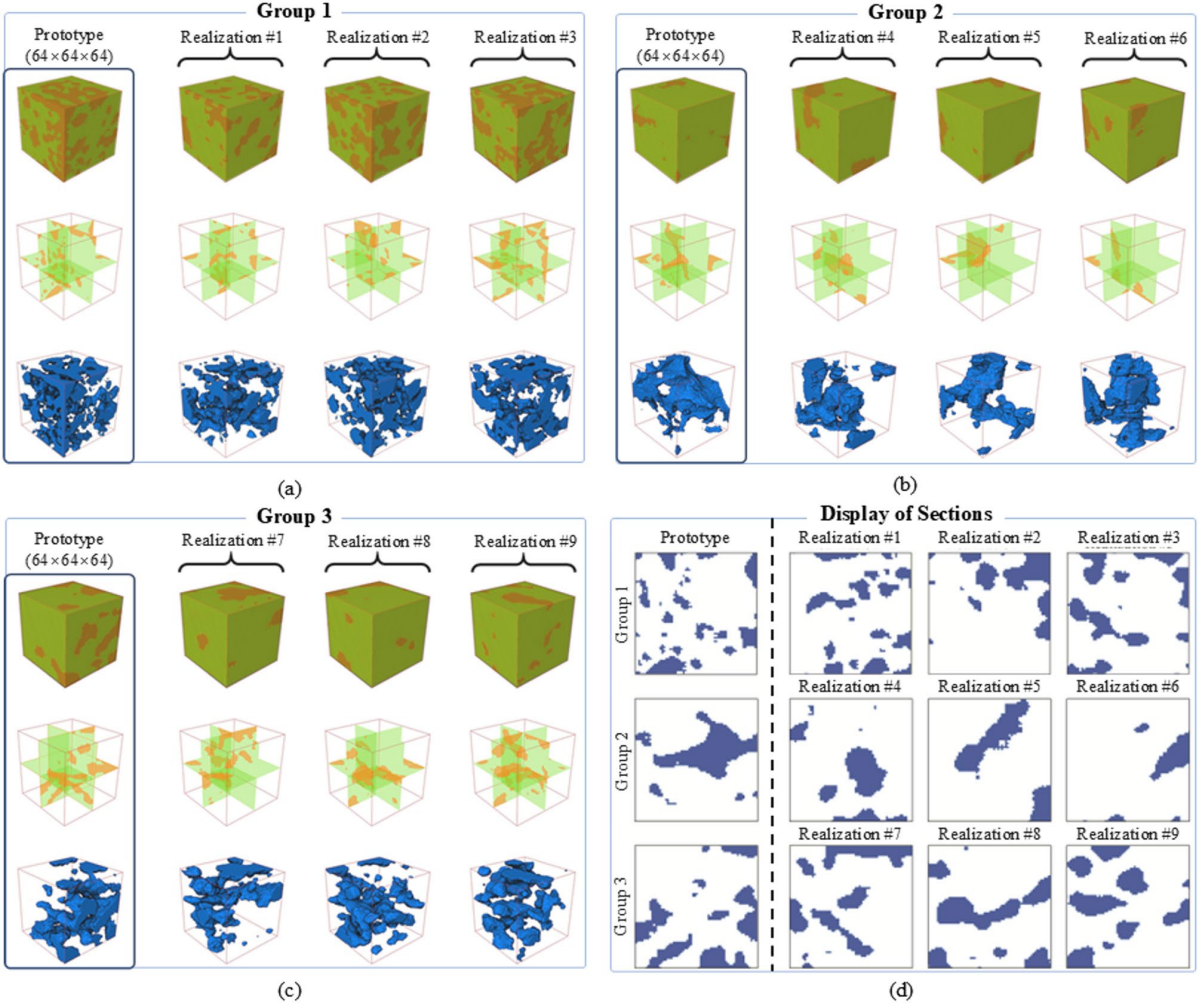
Three groups of training images, each of size 64 × 64 × 64, are used as real data. Three random realizations, reconstructed by the WGAN-GP framework, are generated by sampling 200 random values from a Gaussian distribution (0, 1). These realizations are depicted in 3D formations in panels (**a**–**c**). (**d**) shows a set of 2D cross-sectional views along the middle *z*-direction of each training image and its corresponding realizations. In all figures, the pore space is represented by a dark color. Cited from Zhu & Hu (2024)^47^.

Figure 3 illustrates three randomly chosen realizations for each of the three groups of real gravel soil models, which were randomly selected from the training set with a size of 64 × 64 × 64 *voxels*. The figure showcases different instances or examples of the real models, providing a visual representation of the variability within each group. The realizations in Fig. 3 appear to exhibit a significant similarity to the real soil model. The generated models closely resemble the characteristics and patterns observed in the real soil model, indicating that the training process has been successful in capturing the essential features of the original data. This similarity suggests that the trained models are capable of generating realistic and representative samples that align with the properties of the real gravel soil model.

A dozen methods are used to evaluate the generated results, i.e., multiple dimensional scaling (MDS)^26–28^ and morphological descriptor, i.e., two-point probability function^29^, two-point cluster function^30^ and variogram^16^, respectively. Additionally, an evaluation of the validity of the adopted methods can be obtained by comparing the flow behavior of the generated samples with that of actual samples. The reason for adopting these approaches is explained as follows.

#### Multi-dimensional scaling (MDS) inspection

The quality of the generated results can be evaluated using various methods proposed by numerous researchers^25–34^, including the multi-scale structural similarity index measure (MS-SSIM)^10^, mean structural similarity index measure (SSIM), Multi-Dimensional Scaling (MDS), two-point correlation function, and others. Karras et al. (2018)^32^ proposed that directly using the Earth Mover’s Distance (Wasserstein distance) to evaluate the structural similarity of the generated model and the training model can achieve a better evaluation effect. This method, called multi-dimensional scaling (MDS) approach based on multi-scale sliced Wasserstein distance (MS-SWD), was proposed and successfully utilized to visualize the process of bringing the data distribution of the generated data closer to the actual (training) data. Another, the MS-SSIM or SSIM approach is not suitable for evaluating the detailed structures and realism of generated porous media samples, which used to apply the morphological descriptor^33^, such as two-point correlation function^34^, to yield better evaluation results. By visually observing the simulation results of flow behavior in porous media, it is possible to assess the similarity between the generated samples and training samples and even the validity of the WGAN-GP method. This method provides an intuitive way to observe characteristics such as flow behavior and permeability, in order to determine the realism and accuracy of the generated samples. Of course, relevant hydraulic parameters and other indicators need to be considered in the evaluation process.

**Fig. 4:**
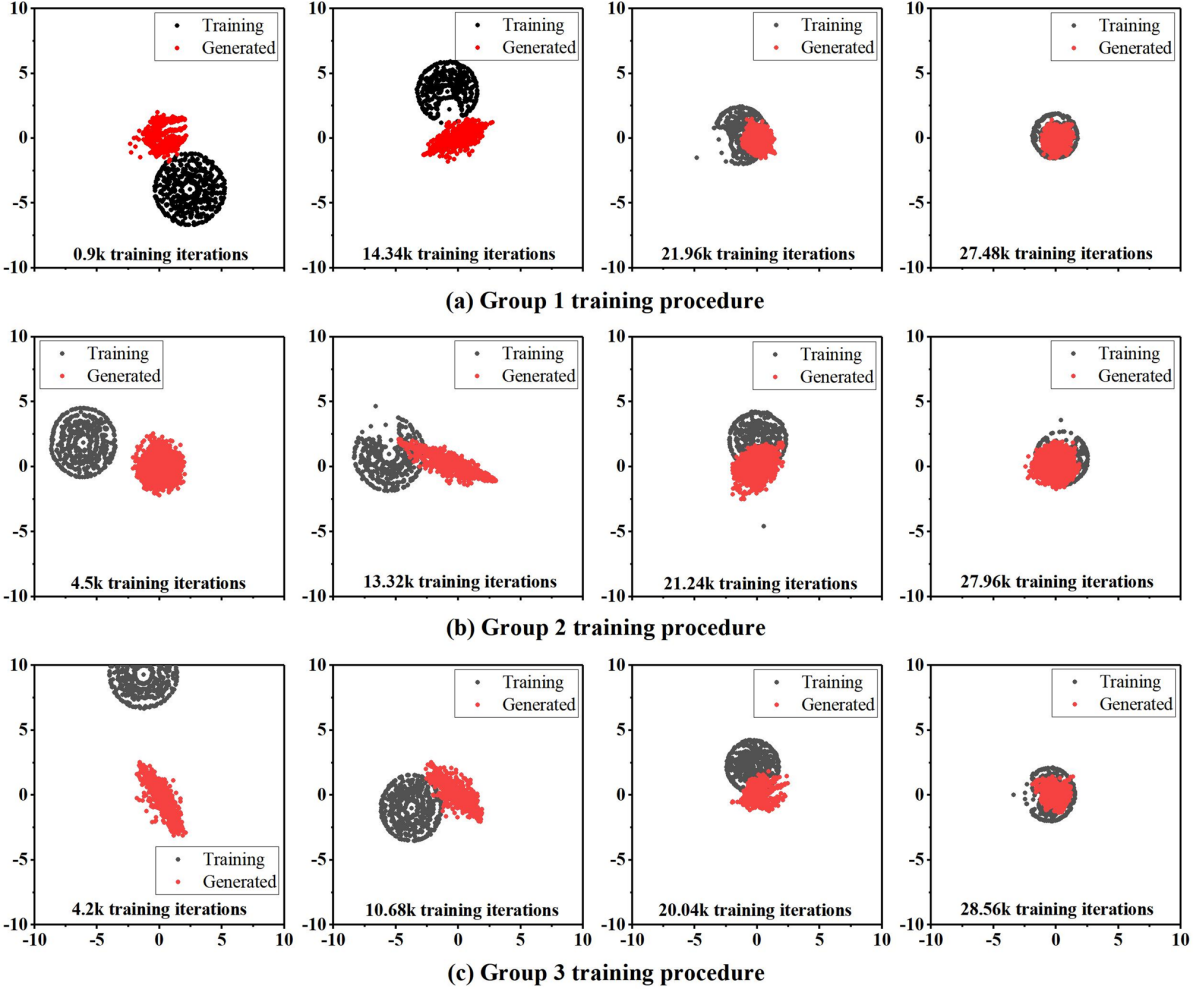
MDS plots of the relationship between the generated gravel soil models (red) and the training gravel soil models (black) in 2D space at different iterations of alternative training, for three groups of training set from (**a**–**c**). As the training progress, the generated gravel soil model distribution gets closer to the training gravel soil model distribution, but after different training iteration steps to get to the optimal of the relationship between these two distributions. (**a**) 27.48k for Group 1; (**b**) 29.64k for Group 2 and (**c**) 27.96k for Group 3.

Multi-Dimensional Scaling (MDS) can be applied to evaluate the WGAN-GP generated model, helping to map the generated images into a lower-dimensional space for visualization and comparison in based on the modified Hausdorff distance^16^. The steps for analyzing WGAN-GP results using MDS are as follows:

1. Train the WGAN-GP and generate a set of synthesized images.
2. Apply dimension reduction technique of MDS to reduce the dimensionality of the generated images based on distance matrix.
3. Use MDS to map the reduced-dimensional data into a two-dimensional space.
4. Visualize and analyze the mapped data, such as labeling different categories of images with different colors or symbols, to observe their distribution and similarity in the space.

By using MDS to analyze the generated images from WGAN-GP, we can gain a better understanding of the learning process of the generator, observe the similarities and differences among the generated images, and further improve the generation performance of WGAN-GP. This analysis method helps to intuitively comprehend the outputs of generative adversarial networks and potentially provides insights for model enhancement. The following MDS plots visualization approach can help to observe the performances of GANs as shown in the Fig. 4.

Three types of gravel soil samples were trained using WGAN-GP, resulting in the generation of 3D simulated samples. Comparing the MDS distributions of the training data and the generated models for the three types of gravel soil samples at different iteration steps, it can be observed that the training data and the generated models eventually blend together to a point where they are difficult to distinguish. This is the desired outcome of GANs, as they aim to achieve a seamless integration between the real and generated samples.

### Morphological descriptors

#### Two-point correlation function

The morphological descriptors frequently used in literatures, namely, two correlation function *S*_2_^(*i*)^(***r***) and lineal-path function *L*^(*i*)^(***r***)^30,35,36^. These descriptors are widely applied by many researchers^16,33,37^. As they provide a good assessment of the similarity between the generative model and the training set.

The two-point probability function *S*_2_^(*i*)^(***r***) is a statistical measure used to describe the spatial relationship between two points in the same phase *i* (e.g., pore space or solid phase) of a material or system. It quantifies the probability that two points, separated by a distance ***r***, both belong to the same phase. This function provides insight into the spatial structure and connectivity of phases in a material. For a given phase *i*, the two-point correlation function is defined as:

6 $$\:{S}_{2}^{\left(i\right)}\left({\varvec{r}}_{1},{\varvec{r}}_{2}\right)=\langle {I}^{\left(i\right)}\left({\varvec{r}}_{1}\right){I}^{\left(i\right)}\left({\varvec{r}}_{2}\right)\rangle$$

where *S*_2_^(*i*)^(***r***_**1**_, ***r***_**2**_) is a statistical descriptors of random media; The vectors of ***r***_1_ and ***r***_2_ represent the position of finding two randomly-selected points in phase *i*; The indicator function *I*^(*i*)^(***r***) = 1 if ***r*** belongs to phase *i* and is zero otherwise; $\:\langle \cdot \rangle$ represents the volume average.

Lu and Torquato^35^ introduced the so-called lineal-path function *L*^(*i*)^(***r***), which describes the probability that a random line of length ***r*** (the “path”) remains within the phase *i* (e.g., void or solid) of the material. This function is a measure of the extent to which the material is connected and how pathways within the phase are structured, so it requires all points that lie on a line between them to fall in the same phase and contains connectedness information along a lineal path. Formally, it can be written as

7 $$\:{L}^{\left(i\right)}\left(\varvec{r}\right)=\langle P\left({\varvec{r}}_{1},{\varvec{r}}_{2}\right)\rangle$$

**Fig. 5:**
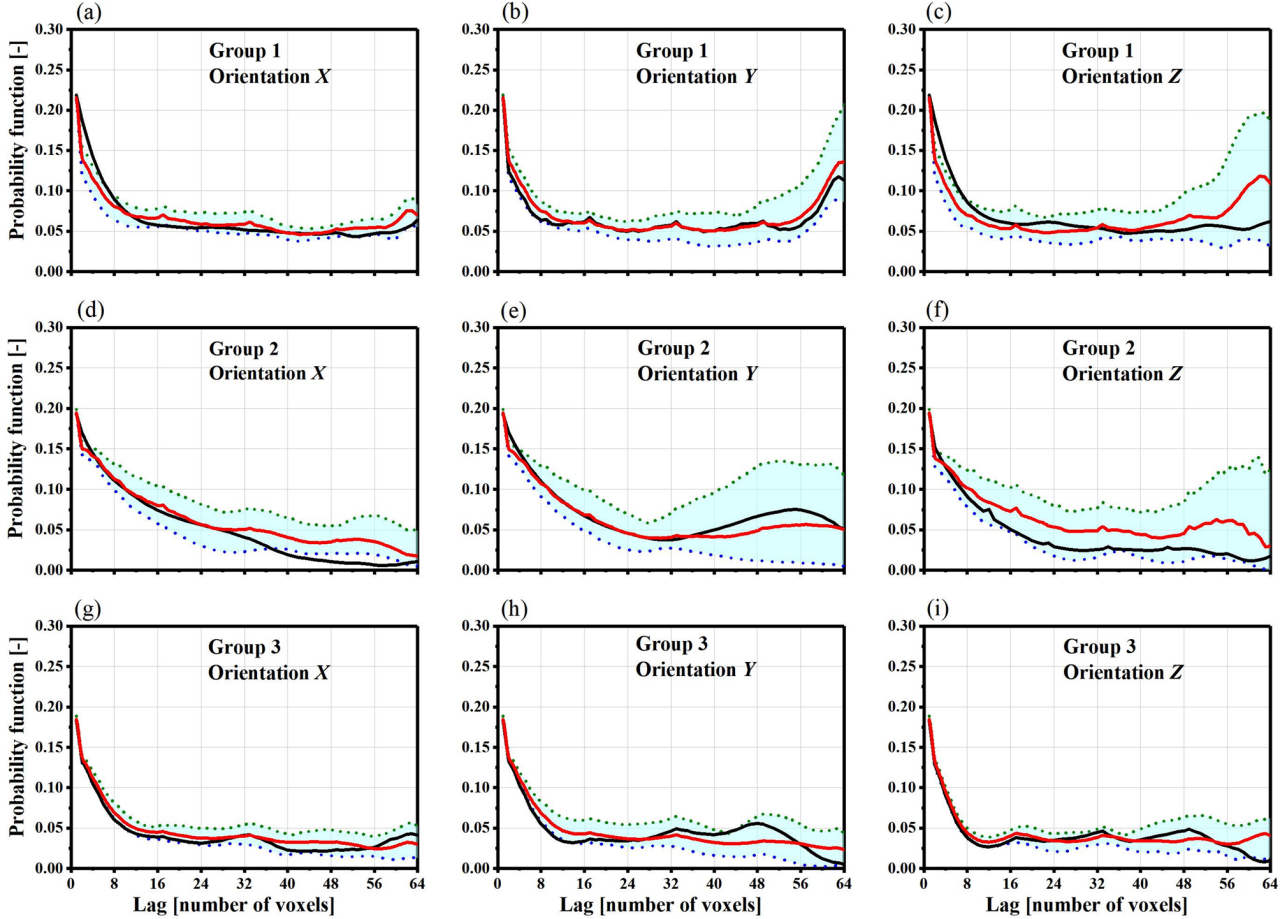
Probability function (PF) of finding porous space at different 3D distances in three gravel soil model groups is calculated for each facies along the *x*, *y*, and *z* axes. The black curves represent PF from a training set (64 × 64 × 64) based on primitive soil prototypes, while the red curves show the mean PF from 14 WGAN-GP realizations (64 × 64 × 64), with dashed lines indicating the minimum and maximum PF values at each distance.

**Fig. 6:**
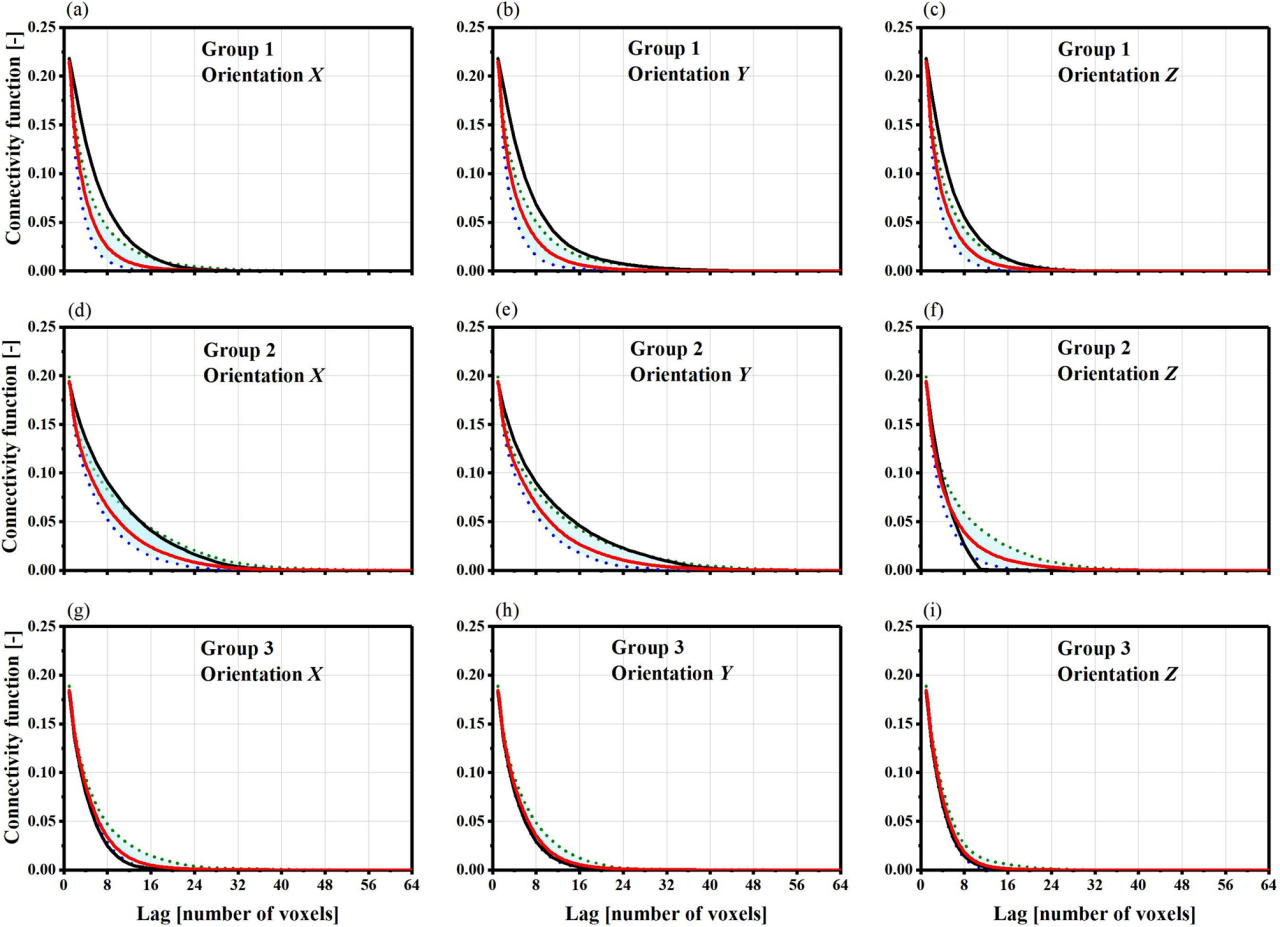
Lineal-Path Function (LF) is used to analyze the pore distribution in 3D gravel soil models along the *x*, *y*, and *z* axes. The black curves show the correlation function (CF) from the original soil models (64 × 64 × 64), while the red curves represent the average CF from 14 WGAN-GP models of the same size. The dashed lines indicate the minimum and maximum CF values at each distance. This comparison helps assess how closely the synthetic models match the real soil distribution.

In this work, we compared the above morphological descriptors of the training dataset and the generated realizations in different direction. Obviously, it is difficult to visually distinguish between the final images generated by WGAN-GP and the training images. In other words, this demonstrates the ability of WGAN-GP to generate images and reproduce the morphology of complex porous materials. If the *S*_2_^(*p*)^(***r***) curves of the generated samples fit well with those of the training samples, it indicates that the generated samples simulate the pore characteristics better, and the same applies to the *L*^(*i*)^(***r***) curves. The both curves of the pore phases for three samples are illustrated in the Figs. 5 and 6, respectively.

The two-point correlation function is a statistical tool used in physics to assess how objects are clustered within a system. It calculates the increased likelihood of finding two objects at a specific distance, relative to a random distribution. In Fig. 5, the probability of encountering porous spaces at various vertical distances (represented by voxel counts) is shown for three groups of generated soil models. These probabilities are compared with those from three groups of primitive soil prototypes of identical size (64 × 64 × 64). The figure offers insights into the porosity and connectivity of the generated porous media, emphasizing the differences between the generated models and the original soil prototypes.

Visually, the generated realizations closely resemble the primitive soil prototypes. The average statistics of the training sets show the best match with the realizations (as seen by comparing the solid red and blue curves) along the *x* direction, with a maximum absolute deviation of 0.05. However, a slight mismatch is noticeable along the *z* direction, where the maximum absolute deviation is 0.22.

The lineal-path function (Fig. 6), or connectivity function, shows a strong agreement between the average statistics of the training sets and the WGAN model realizations (solid red and blue curves) along the *x*, *y*, and *z* axes, with a maximum absolute deviation of about 0.05. The soil prototype exhibited slightly higher connectivity. This comparison suggests that, although the WGAN model realizations are not perfect, they perform better than those generated by other recent algorithms, like Simulated Annealing (SA), in terms of quality. Furthermore, when a large number of model realizations is needed, the WGAN model offers faster performance.

#### Minkowski function

The specific surface area and Euler characteristic are two fundamental concepts in the field of geometric topology. Together with porosity *ϕ*, they are also called the order zero, one and 3rd Minkowski function^38^. They characterize a *d*-dimensional convex body, which forms a detailed description of the complex porous-material construction here, through a series of quantized parameters.

The specific surface area of a convex body in *d*-dimensional space refers to the total surface area per unit volume. It quantifies the extent of surface area relative to the volume enclosed by the convex body. In porous-material constructions, the specific surface area is crucial for understanding properties such as adsorption, reaction rates, and permeability^39^. It is defined as:

8 $$\:{S}_{V}=\frac{1}{V}\int\:dS$$

where integration occurs over the void-solid interface *S*. The specific surface area *S*_*V*_ has dimensions of $\:\frac{1}{length}$ and its inverse allows us to define a characteristic-pore size.

The Euler characteristic is a topological invariant that relates the number of vertices, edges, and faces of a polyhedral surface. In more general terms, for a *d*-dimensional convex body, the Euler characteristic relates the number of *d*-dimensional cells, (*d*-1)-dimensional faces, …, 1-dimensional edges, and 0-dimensional vertices. For more complex shapes, such as those with holes or cavities, the Euler characteristic can be generalized using more advanced definitions, but it still serves as a key measure in understanding the topology of the object.

The Euler characteristic is used to classify different shapes and structures based on their topological properties. It is defined as:

9 $$\chi =V - E\,+\,F$$

where *χ* is the Euler characteristic, *V* is the number of vertices, *E* is the number of edges, and *F* is the number of faces.

The specific Euler characteristic refers to the Euler characteristic per unit volume or per unit area. It is a commonly used parameter because it normalizes the Euler characteristic to account for the size or scale of the object. This adjusted measure allows for a more scale-independent comparison of the topological features of different structures, making it more comparable across different structures. It is defined as:

10 $$\:{\chi\:}_{specific}=\frac{\chi\:}{V}(\text{f}\text{o}\text{r}\:\text{v}\text{o}\text{l}\text{u}\text{m}\text{e}-\text{b}\text{a}\text{s}\text{e}\text{d})\:\text{o}\text{r}\:{\chi\:}_{specific}=\frac{\chi\:}{A}(\text{f}\text{o}\text{r}\:\text{a}\text{r}\text{e}\text{a}-\text{b}\text{a}\text{s}\text{e}\text{d})$$

where *χ*_*specific*_ is the specific Euler characteristic; *V* is the volume of object (for volume-based specific Euler characteristic); A is the surface area of the object (for area-based specific Euler characteristic).

**Fig. 7:**
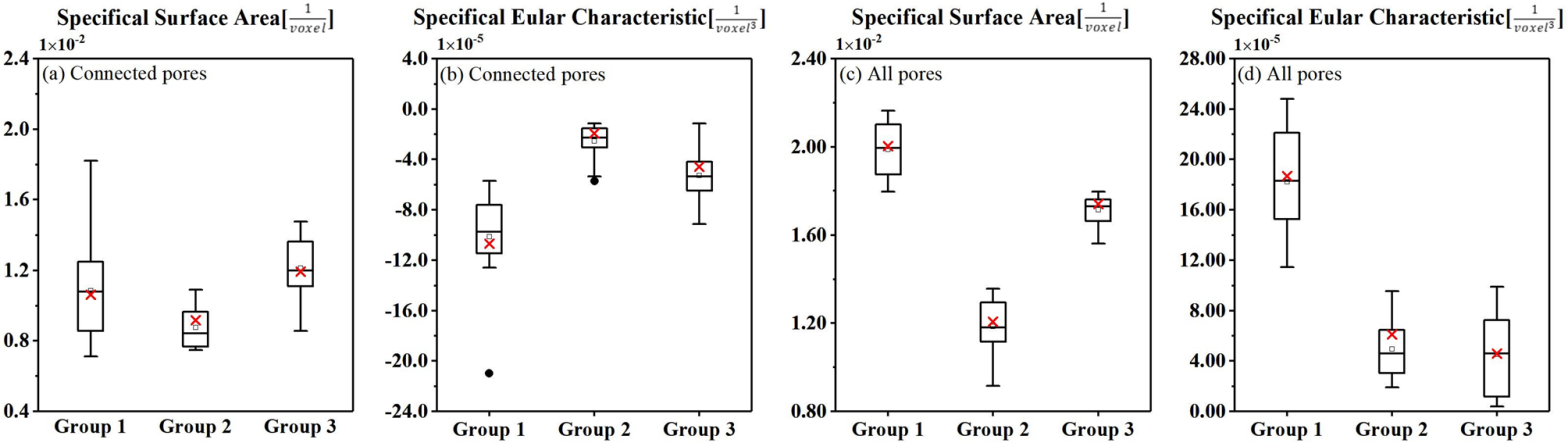
Comparison of specigical surface area and specifical Euler characteristic of every group of realization. They show good agreement that the most of the value of prototype models (red cross) are located at the range of the realization boxes. (**a**,**b**) are for the connected pores; (**c**,**d**) are for the whole pores including the connected pores and the dead pores.

We can acquire the specific surface area and Euler characteristic from the open-source image morphological software library MorphoLibJ^40^. The results of directly computing the two Minkowski functionals are presented in Fig. 7, showing comparable distributions for both the connected pores (Fig. 7a,b) and all pores (Fig. 7c,d) in the training images and the synthetic realizations.

## Hydraulic behavior simulation

### Flow simulation in the porous media

The fluid flow within generated soil model can be analyzed using various numerical simulation techniques, such as computational fluid dynamics (CFD) or pore-scale modeling. These methods involve solving the governing equations of fluid dynamics, such as Navier-Stokes equations, within the complex geometry and topology of the soil model. Flow in the free channel is described by the stationary, incompressible Navier-Stokes equations as follow.

11 $$\:\begin{array}{c}\rho\:\left(\mathbf{u}\bullet\:\nabla\:\right)\mathbf{u}=-\nabla\:\bullet\:\left[-p\mathbf{I}+\mu\:\left(\nabla\:\mathbf{u}+{\left(\nabla\:\mathbf{u}\right)}^{T}\right)\right]\\\:\nabla\:\bullet\:\mathbf{u}=0\end{array}$$

where *µ* denotes the dynamic viscosity (Pa‧s), **u** refers to the velocity in the open channel (m/s), ρ is the fluid’s density (kg/m^3^), and p is the pressure (kPa).

**Fig. 8:**
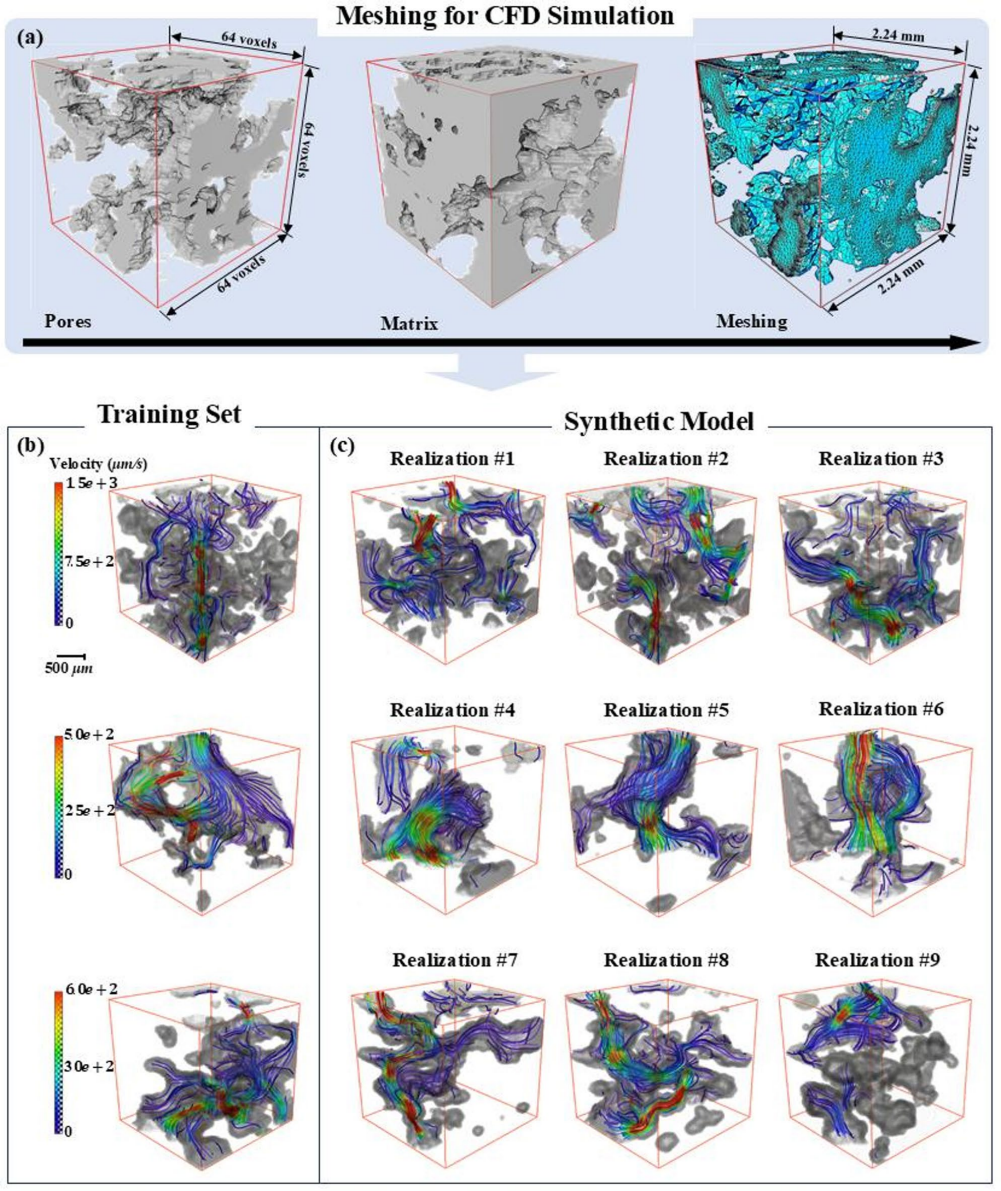
3D CFD simulation of fluid flow diagrams: (**a**) meshing process including pores generation, matrix space and conforming mesh (#Nodes: 31596/# Tetrahedrons: 88838) in pore space (**b**) CFD simulations of real fluid flow in the training set with colored flow lines indicating changes in velocity. (**c**) CFD simulations of synthetic model. The diagrams compare the real fluid flow in the training set with the simulated flow in the model. Partially cited from Zhu & Hu^47^.

By simulating the fluid flow within the generated soil model, it is possible to gain insights into the transport properties of the porous media, including permeability, porosity, and tortuosity. The results of these simulations can be used to design and optimize engineering applications that involve fluid flow in porous media, such as groundwater management, petroleum engineering, and geothermal energy extraction. Inhere, a generated soil model is considered as the porous media, in which the flow field in detail can be feasible by numerical simulations at the pore-scale. In this work, the absolute permeability is estimated with Avizo 2020. Figure 8a illustrates an example of mesh generation for an original or generative model with 31,596 nodes and 88,838 tetrahedra, which numbers are depended upon the pore space realized as simulation domain. Overall, a series of generative models, which simulate the three groups of micro-structures as training sets in context, are over 50 in total. The portion depicted in Fig. 8b, c represents only a subset of these examples.

Table 1 lists the parameters used in the simulated model of all realizations. The accuracy and reliability of the fluid flow simulations strongly depend on various factors, such as the resolution and quality of the generated soil model, the complexity of the fluid-solid interactions, and the assumptions and approximations made in the numerical methods. Therefore, careful validation and verification of the simulation results are crucial to ensure their validity and applicability to real-world scenarios.

**Table 1:** List of parameters used in the fluid-flow simulation model of all realizations.

Property	Value
Dynamic Viscosity *µ* [Pa‧s]	1.0 × 10^− 3^
Density *ρ* [kg/m^3^]	1000
Porosity *ε*_*p*_ of group 1 [%]	21.8
Porosity *ε*_*p*_ of group 2 [%]	19.1
Porosity *ε*_*p*_ of group 3 [%]	18.2
Cross-section of a Model *S* [mm^2^/voxels × voxels]	5.0/64 × 64
Length along flowing direction *L* [mm/voxels]	2.24/64
Input pressure *p*_*in*_ [kPa]	120.0
Output pressure *p*_*out*_ [kPa]	100.0

Based on the pore-scale simulation results, it is often a practical approach to utilize macroscopic approaches that rely on averaged quantities of the porous structure, such as porosity and permeability, to address issues involving sophisticated scale effects. The following expression is used.

12 $$\:\kappa\:=\frac{Q}{S}\mu\:\frac{L}{{\Delta\:}p}\:\:\:or\:\:{k}_{h}=\kappa\:\frac{\rho\:g}{\mu\:}$$

where *Q* is the global flow rate passing through the porous medium (m^3^‧*s*^-1^); *S* is the cross section of the realization the fluid goes through (mm^2^); $\:\kappa\:$ is the intrinsic permeability (µm^2^ or Darcy); *k*_*h*_ is the hydraulic conductivity (cm/s); *p*_*in*_, *p*_*out*_ is the pressure in the inlet and outlet (kPa), respectively; *L* is the length of the realization in the flow direction (mm).

### Hydraulic behaviors analysis

The velocity field in each realization and corresponding training set are shown in Fig. 8. Characteristics for the flow in a realization are areas with high and areas with low velocity. In some areas the flow also stagnates. Figure 8 also shows the velocity field of different realizations within the same group. It is observed that each realization within the group exhibits similar seepage characteristics. This suggests that within a specific group, the flow patterns and seepage behavior remain consistent across different realizations. The similarity in the velocity field indicates that the fluid flow through the porous medium follows a similar pattern, regardless of the specific realization.

This behavior is characterized by the permeability, which conversions may be derived to calculate from intrinsic permeability $\:\kappa\:$ to hydraulic conductivity *k*. Generally, there is the expression for their relationship.

13 $$\:k\left(incm/s\right)=\kappa\:\left(in\:{\mu\:m}^{2}\right)\times\:9.7\times\:{10}^{-4}$$

Intrinsic permeability refers to the ability of the porous medium to allow fluid flow, while hydraulic conductivity takes into account both the porous medium and the fluid properties. Intrinsic permeability measures the ease of fluid movement, whereas hydraulic conductivity defines the actual fluid flow rate given a pressure difference.

**Table 2:** Statistics of hydraulic conductivity of prototype and realization models.

No.	k_x_ [cm/s]		k_y_ [cm/s]		k_z_ [cm/s]	
	Prototype	Avg. of realizations	Prototype	Avg. of realizations	Prototype	Avg. of realizations
Group 1	0.98 × 10^− 2^	1.32 × 10^− 2^	0.91 × 10^− 2^	1.55 × 10^− 2^	1.15 × 10^− 2^	1.43 × 10^− 2^
Group 2	5.44 × 10^− 2^	4.40 × 10^− 2^	4.48 × 10^− 2^	4.44 × 10^− 2^	2.02 × 10^− 2^	2.29 × 10^− 2^
Group 3	2.59 × 10^− 2^	3.00 × 10^− 2^	2.71 × 10^− 2^	2.91 × 10^− 2^	1.15 × 10^− 2^	1.30 × 10^− 2^

**Fig. 9:**
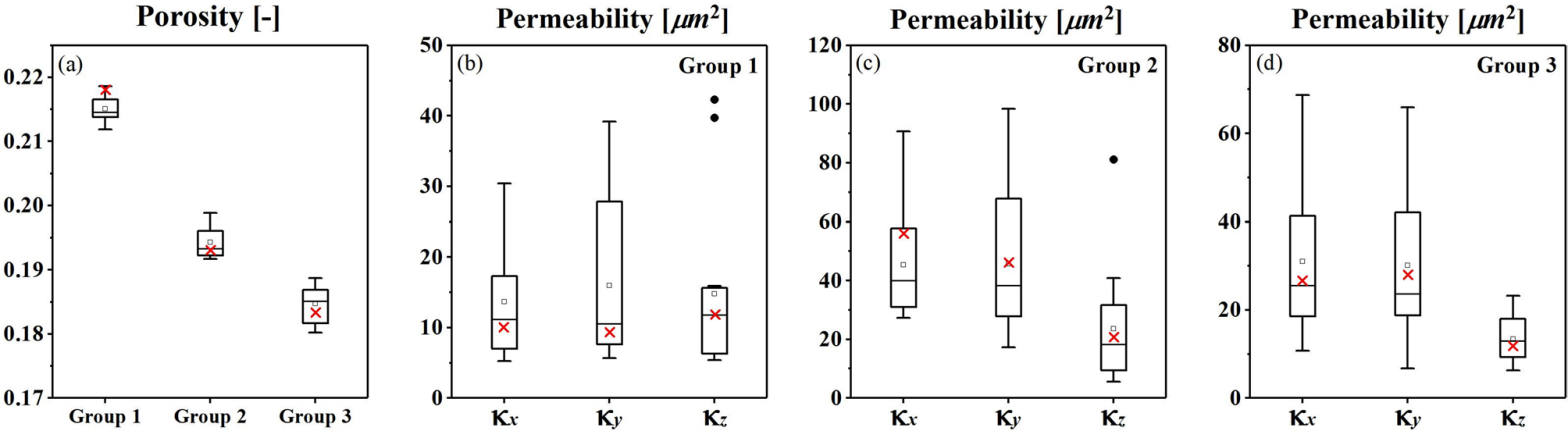
Comparison of porosity, absolute permeability of three direction of every group of realization. They show good agreement that the most of the value of prototype models (red cross) are located at the range of the realization boxes.

Table 2 lists the statistical intrinsic permeability of each group of soil model based on fourteen realizations. The corresponding hydraulic conductivities are converted based on the Eq. 13. Figure 9 illustrates the box chart of the geometrical parameters of 14 realizations of generated gravel soil model, including statistics of porosity (Fig. 9a) and permeability (Fig. 9b–d).

Figure 9b–d illustrates a lognormal distribution of absolute permeability, demonstrating that the permeability of the generated model aligns closely with that of the prototype. Acturally, according to the known formula regarding the relationship between permeability and porosity, i.e. Kozeny -Carman formula. There is a power law relationship between the two parameters:

14 $$\:k=\frac{{d}_{h}^{2}}{180}\frac{{{\epsilon\:}_{p}}^{3}}{{\left(1-{\epsilon\:}_{p}\right)}^{2}}$$

where: dh is the equivalent grain size of porous materials. Related content can be found in reference^1,3^.

**Fig. 10:**
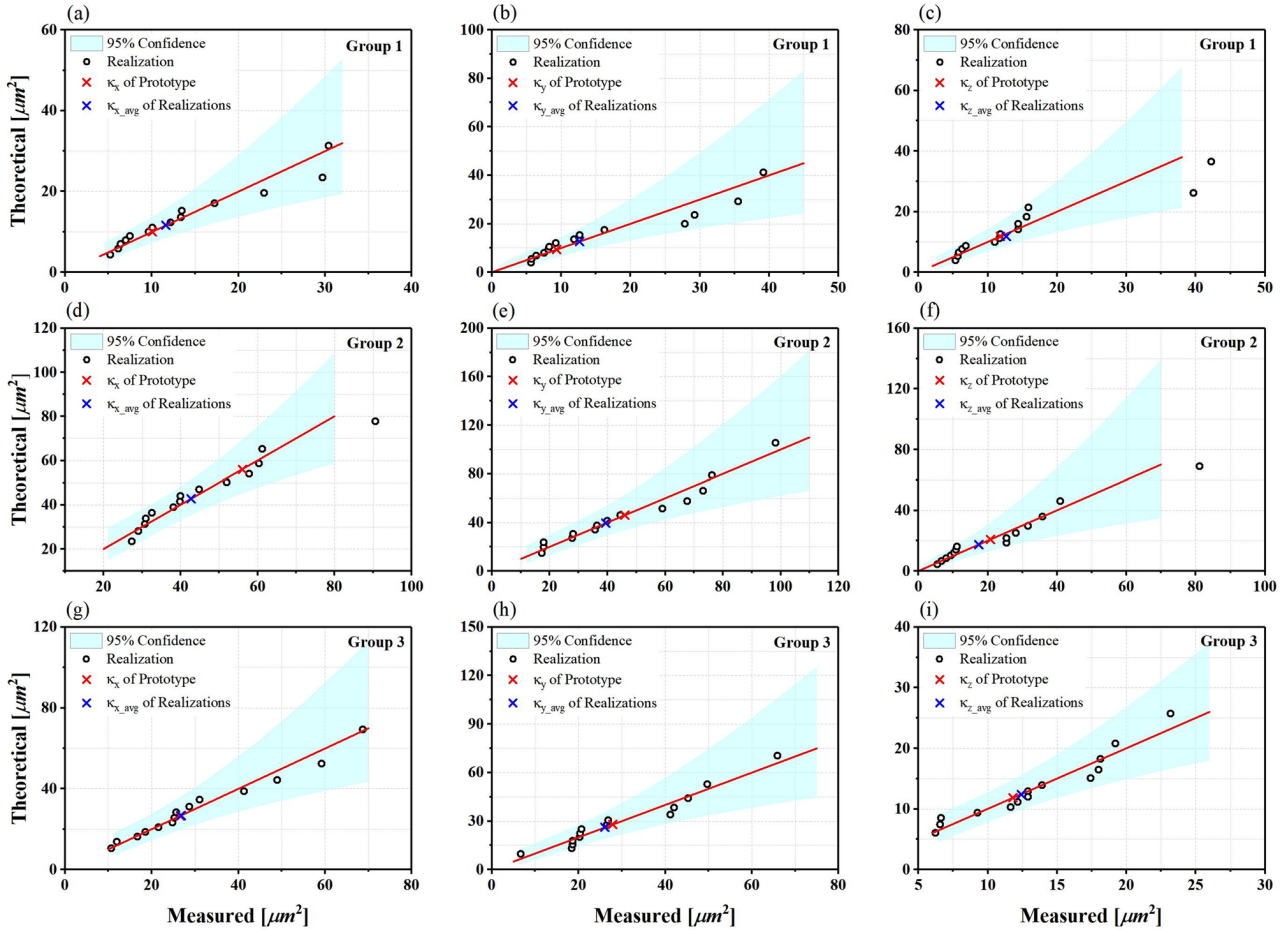
Q-Q plot of the permeability statistics of the 14 realizations based on the hypothesis of lognormal distribution. (a–c) Group 1; (d–f) Group 2; (g–i) Group 3. Red cross signs represent the permeability of gravel soil prototype, and blue ones the permeability average of 14 realizations.

Based on Table 2, hypothesis tests were conducted to identify the best distribution for the permeability of each realization group. A Q-Q plot was created to evaluate how well or goodness the lognormal distribution fits the 14 separate realizations. The result of the analysis shows that there is fitness in different extent between the lognormal distribution and each of the 14 realizations for three groups, respectively. The Q-Q plots are shown in Fig. 10.

A Q-Q plot is a useful tool for examining the fit of a dataset to a specific probability distribution. By comparing the observed quantiles with the expected quantiles from the chosen distribution, the Q-Q plot can reveal whether the data follows the theoretical distribution or not. Furthermore, what the strategy of generation based on WGAN-GP is good or not. A straight line on the Q-Q plot indicates a goodness-of-fit between the data and the distribution, while deviations from the line suggest departures from the expected distribution. From the Q-Q plot of the 14 realizations based on the hypothesis of lognormal distribution resulted in a straight line, indicating a good fit between the lognormal distribution and the data. Particularly, the hydraulic conductivity of the realizations of group 1 and 3 is perfect fitness between the value of prototype and Avg. of realizations. It can be seen that there are some discrepancies on the fitness of group 2, but it can be ignored within the same order of magnitude – i.e., the range of 10^− 2^ cm/s. This indicates basically the seepage ability of fine sand. This implies that the behavior of generation by WGAN-GP can be an appropriate model to producing the lognormal distribution by describing the behavior of the data in each of the 14 realizations.

## Discussion

Our results demonstrate that our proposed WGAN-GP approach works well. Figure 3 shows the comparison between the real prototype and generated realizations. By examining both 3D models, the generated microstructure closely resembles the real microstructure, making it hard to tell them apart. By comparison between the morphology parameters of the generated model and corresponding soil prototype (Fig. 7), the results showed quantitatively that the morphological characteristics of the generated model closely resembled those of the soil prototype. It suggests that the generator accurately captured the key morphological features of the natural soil, and is capable of emulating the complex micro-geometry of the soil with diverse and realistic shapes of voids and matrix of generated microstructures. This presents a notable advancement for certain gravel soils, which pose challenges in obtaining intact in-situ samples, as well as for rocks drilled at considerable depths, where the associated costs are prohibitively high for study purposes.

**Fig. 11:**
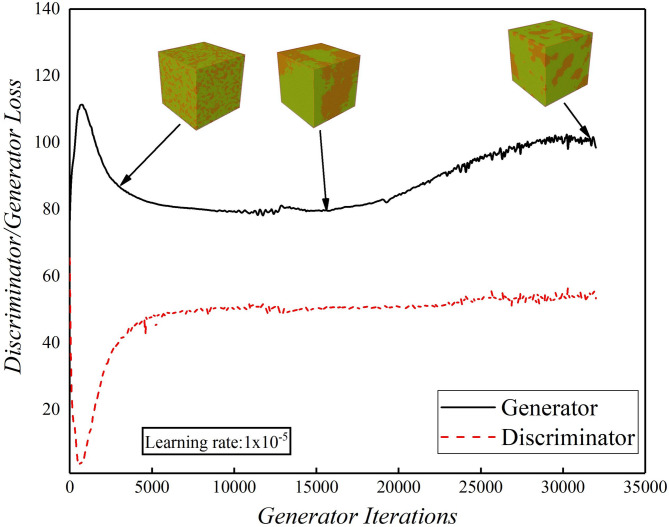
Value of the cost function, i.e., loss of the discriminator and generator for the WGAN-GP trained on the realization #1 of training set (Group 1). The 3D models shown were derived from the random number seed satisfied with Gaussian distribution, i.e., *z* ~ Gaussian (0,1), showing the process of evolution during training. It can be seen that the 3D model with very low quality and noise image was evolving to one with coarse structures in 15,000 iterations, and finally similar to the training set (Group 1) from soil prototype after 30,000 iterations. Learning rates were kept in a small value of 10^− 5^.

The MDS plot (Fig. 4) illustrates the process of earth-moving and eventual convergence. Figure 11 depicts the training curve for the realization #1 of a training set of Group 1. At the beginning, the generator loss function [as described in Eq. (3)] is notably high, and no discernible structural components are present in the generated samples. However, as the generator loss function undergoes a substantial reduction, initial structures start to emerge. The quality of image reconstruction shows significant enhancement with an increasing number of generator iterations, even though it cannot be directly correlated with the loss function of the generator. This is evident from the observation of a rise in generator loss towards the end of the training process, despite a considerable improvement in image quality. During the training process, manual inspection of synthetic realizations was performed to ensure convergence. The training was stopped based on the evaluation of two-point statistics as shown in Figs. 5 and 6.

Figure 8 provides a detailed visualization of the hydraulic simulation results, showcasing the fluid flow within the connective pores for three distinct groups of training sets and their respective realizations. It is evident that the actual velocity varies within the tortuous flow pipe, with some areas experiencing high velocity while others have lower velocity. Similarly, the flow lines appear concentrated in certain regions and sparse in others. These disparities in actual velocity across different parts of the gravel soil can lead to varying degrees of erosion. Higher flow rates tend to result in easier erosion of gravel soil particles, while more concentrated streamlines increase vulnerability to soil instability through piping erosion^3,4^. The first group of gravelly soil is particularly susceptible to erosion, as it tends to develop into quick-sand or flow-soil due to piping erosion and exhibits unstable seepage. The other two groups are also prone to piping erosion but remain stable^2,6^. Please refer to our recently published paper by Zhu et al. (2023)^1^ for further details on our research. Notably, the hydraulic permeability of every group counted statistically on Table 2 indicates a flow rate on the order of ×10^− 2^ cm/s, which aligns with the results obtained using classical criteria and general engineering experience.

Additionally, we validated the microstructures generated by WGAN-GP using several physics-based metrics, including porosity (*ϕ*), specific surface area (SV), specific Euler characteristic (χ_V), and permeability (κ). The validation results, illustrated in Figs. 9 and 10; Table 2, indicate that the physical properties of the generated models generally align well with those of real models. Both the original and generated samples have porosity values ranging from 0.18 to 0.22. However, their overall connectivity is poor, as reflected by the positive values of specific Euler characteristics (Fig. 7). Moreover, the permeability analysis for seepage, which mimics Darcy’s law, consists of quantitative measures derived from CFD analysis based on the Navier-Stokes equations. Notably, the proposed 3D-WGAN-GP generator produces synthesized microstructures with permeability values ranging from 1.36 × 10^–11^ to 4.54 × 10^–11^ m^2^ (x-axis), 1.60 × 10^–11^ to 4.58 × 10^–11^ m^2^ (y-axis), and 1.34 × 10^–11^ to 2.36 × 10^–11^ m^2^ (z-axis), which closely resembles the permeability range of the real soil prototype, covering 1.01 × 10^–11^ to 5.61 × 10^–11^ m^2^ (x-axis), 0.94 × 10^–11^ to 4.62 × 10^–11^ m^2^ (y-axis), and 1.19 × 10^–11^ to 2.08 × 10^–11^ m^2^ (z-axis). Additionally, the hydraulic conductivity distribution of the generated realizations with synthesized microstructures follows a log-normal distribution (Fig. 10), suggesting that our 3D-WGAN-GP code exhibits a tendency to generate smaller permeability values.

Comparing our results with those of Mosser et al. (2017)^38^, we find that Berea sandstone exhibits similar characteristics—such as porosity, specific surface area, and Euler characteristics—as the soil specimens in group 2. Additionally, both Berea sandstone and the group 2 specimens share an image size of 64^3^ voxels. However, there is a significant discrepancy in permeability, which is difference of two orders of magnitude. The commonly used Kozeny-Carman permeability formula states that permeability is influenced not only by porosity but also by the equivalent diameter of the particles. Upon detailed comparison, we observed that our three samples are comparable to the Berea sample^38^ in terms of specific surface area, Euler characteristics, and porosity. However, there is a notable difference in permeability: while the Berea sample has a permeability around 10^− 13^ m^2^, our samples range between 10^− 10^ m^2^, resulting in a difference of three orders of magnitude. This variation is due to the uneven nature of our samples, with larger particles increasing their permeability. Our generative model successfully captures this distinction, unlike the more uniform Berea samples. This discrepancy may imply different fluid flow characteristics and responsiveness to hydraulic pressures, which could be crucial for applications in hydrogeology and soil mechanics.

The limitation of the proposed approach for modeling gravel soil, particularly the small scale (64^3^-voxel) patches used in the soil model that fail to capture the geometric characteristics of coarse grains. Although fine grains dominate the hydraulic properties, the hydraulic effect of coarse particles is significant. To address this issue, the paragraph discusses expanding the training patches to larger sizes, such as 128^3^-voxel or 192^3^-voxel, to capture more detail, but acknowledges that this will increase hardware and software requirements and training time. A feasible strategy suggested is to begin with 2D faces of the soil prototype, allowing for larger training sets and optimizing training time by calculating voxel values in other directions using a geostatistical variogram function. This approach may lead to improved efficiency, as demonstrated in existing literature. The limitation mentioned above arises from the scarcity of CT image data, and obtaining a large number of samples suitable for large-scale training is a challenge due to financial constraints. To overcome this, the only feasible approach is to reduce the sample size, which allows for generating a large quantity of training datasets, especially for 3D core samples.

Currently, the majority of application cases of GAN-based generation primarily focus on processing binary structure grayscale images of pores and matrix^9,32,38,43,44^. In the context of limited access to large quantities of high-quality data, achieving superior detection performance and applying it more effectively to related fields has led to the emergence of deep learning algorithms based on few-shot learning as a prominent research topic within the domain of artificial intelligence. Few-shot machine learning seeks to leverage prior knowledge to address the issue of insufficient training data caused by data scarcity, enhance the model’s generalization capabilities across new categories, and significantly reduce both the cost and complexity associated with acquiring and constructing datasets^46^.

Laloy et al.^33,45^ developed a method to quickly generate 2D or 3D models from a channelized aquifer training image using their proposed Spatial Generative Adversarial Network (SGAN). A key feature of SGAN is its low-dimensional parameterization, which allows for efficient probabilistic inversion with Markov Chain Monte Carlo (MCMC) methods. This machine learning approach is based on multi-point geostatistical (MPS) methods for inversion parameters. Unlike traditional methods, SGAN starts with a prior probability variable (θ) instead of a uniform distribution latent variable (Ƶ), reducing training time to just 3–12 h. In comparison, our approach to generating porous media takes longer to train than probabilistic MPS methods. However, if we first focus on training random function parameters before adding the adversarial network, we can reduce generation time. This method offers a more efficient way to generate synthetic images, though applying MPS to porous media remains challenging, despite its success in channel-like models^41–43^.

Song et al.^16^ used GANs for geological facies modeling with both progressive and conventional training workflows, evaluating the performance of the two approaches. This solution addresses challenges in geological modeling related to size and scale. In a follow-up study, Song et al.^44^ discussed the limitations of the MPS method, which uses statistical data from a narrow range of training images, resulting in an incomplete representation of geological features over a larger area. MPS can perform poorly in complex geological structures or dense well-point data, particularly in cases involving intermittent channel facies. Conditional GANs may offer a better solution for small-scale micro-porous modeling, compared to MPS, which is more suited for large-scale geological modeling.

The aforementioned approach can be viewed as an effective strategy for obtaining large-scale generative models through machine learning, leveraging an existing set of prior MPS models. This approach bears similarity to few-shot learning, effectively addressing the challenges posed by insufficient training data. The WGANs method proposed in this study enables the design of more intelligent models by integrating the aforementioned few-shot process with domain knowledge and prior information. In the context of few-shot learning, existing domain knowledge—such as the physical properties of rock cores, the geometric distribution of soil, and other relevant factors—is utilized to guide the design of the model structure, thereby enhancing the model’s capability to perform feature extraction and learning. In scenarios characterized by data scarcity, the integration of techniques such as transfer learning, data augmentation, generative adversarial networks, and meta-learning can significantly bolster the model’s detection performance on small sample datasets. These advanced methodologies not only mitigate the cost and difficulty of data acquisition but also enhance the model’s generalization ability across novel categories, thereby offering substantial support for real-world applications.

## Conclusions

An innovative 3D training-image approach based the so-called Earth-Mover (EM) or Wasserstein distance for a complex gravel soil is presented. Direct 3D convolution kernel is used to extract image features of porous microstructure more effectively. Average 9 h of training time is a good score for a basic configuration of laptop. By matching important statistical and physical parameters, such as two-point statistics, porosity, specific surface and Euler characteristic, the WGAN-generated synthetic images provide a reliable representation of real-world porous media. This enhances our understanding of the flow and transport phenomena within these materials and aids in the development of accurate predictive models. The results of the seepage simulation for each realization are consistent with the established classical criteria for evaluating the erosion stability of gravel soil. By quantitatively evaluating the absolute permeability, we have shown that the synthetic realization images generated by the WGANs model are able to match key characteristic statistical and physical parameters of these soil prototype. The behavior of generation of the WGANs is more incline to a lognormal distribution of absolute permeability of realizations. Main attempts for future research include how to identify more details for a grading gravel soil through expanding step-by-step the region of 3D patches as training set, or conversion a large region 2D training-image to 3D synthetic realization. Meanwhile, using color level to distinguish the particle size grading for gravel soil is also a significant extension to be made to make the WGANs framework more practical and usable. Currently, the main limitations of this study primarily lie in generating larger-scale models (such as 128³, 256³, or even 400³) with a limited number of samples. Future developments will focus on the following directions: (1) Few-shot deep learning combined with MPS model, (2) Physical modeling combined with machine learning based on prior knowledge.

## Data Availability

The code and plotting data for WGAN-GP in the study are freely available at GitHub via https://github.com/Zhu-Bi/Gravelsoil_GAN (Zhu & Hu, 2024). The μ-CT image data for ML learning in the study are freely accessible in Figshare (https://figshare.com/articles/figure/CT_image_for_gravel_soil/25665360) (CC BY 4.0) (Zhu & Hu, 2024).
